# ACTH and gonadotropin deficiencies predict mortality in patients treated for nonfunctioning pituitary adenoma: long‐term follow‐up of 519 patients in two large European centres

**DOI:** 10.1111/cen.13141

**Published:** 2016-07-25

**Authors:** Michael W. O'Reilly, Raoul C. Reulen, Saket Gupta, Claire A. Thompson, Rosemary Dineen, Eirena L. Goulden, Gabriella Bugg, Harriet Pearce, Andy A. Toogood, Neil J. Gittoes, Rosalind Mitchell, Christopher J. Thompson, John Ayuk

**Affiliations:** ^1^ Institute of Metabolism and Systems ResearchUniversity of BirminghamBirminghamUK; ^2^ Centre for Endocrinology, Diabetes and MetabolismBirmingham Health PartnersBirminghamUK; ^3^ Department of EndocrinologyUniversity Hospitals BirminghamBirminghamUK; ^4^ School of Health and Population SciencesUniversity of BirminghamBirminghamUK; ^5^ Division of EndocrinologyBeaumont Hospital and RCSI Medical SchoolDublin 9Ireland; ^6^ Department of NeurosurgeryUniversity Hospitals BirminghamBirminghamUK

## Abstract

**Context and Objective:**

Nonfunctioning pituitary adenomas (NFPAs) are the most common subtype of pituitary tumour. Hypopituitarism is observed in NFPAs due to tumour‐ or treatment‐related factors and may increase mortality risk. Here, we analysed the associations of hypopituitarism, hormone replacement and mortality in a large NFPA cohort derived from two large European centres.

**Design, Setting and Participants:**

Case note review of all patients treated for NFPA in University Hospitals Birmingham and Beaumont Hospital Dublin between 1999 and 2014 was performed.

**Main Outcome Measures:**

Clinical presentation, treatment strategies, pituitary function and vitality status were recorded in each patient. A multivariate Cox regression model was used to examine the association between hypopituitarism, hormone replacement and premature mortality.

**Results:**

A total of 519 patients were included in the analysis. Median duration of follow‐up was 7·0 years (0·5–43). A total of 81 deaths were recorded (15·6%). On multivariate analysis, adrenocorticotropic hormone (ACTH) and gonadotropin (Gn) deficiencies were associated with an increased relative risk of death (OR 2·26, 95% CI 1·15–4·47, *P* = 0·01 and OR 2·56, 95% CI 1·10–5·96, *P* = 0·01, respectively). Increased hydrocortisone (HC) (*P*‐trend = 0·02) and lower levothyroxine (LT4) doses (*P*‐trend = 0·03) were associated with increased risk of death. Mortality increased with the degree of pituitary failure observed (*P*‐trend = 0·04).

**Conclusion:**

ACTH and gonadotropin‐deficient patients have higher mortality rates compared to those with intact hormonal axes. Excessive HC and suboptimal LT4 replacement may also increase risk of death. Complex associations between hormone deficiency and replacement underpin the increased mortality risk in NFPA patients.

## Introduction

Nonfunctioning pituitary adenomas (NFPAs) are benign tumours which do not cause clinical syndromes of pituitary hormone hypersecretion.^1^ They constitute one‐third of all pituitary neoplasms^2^, ^3^ and have a prevalence of approximately 22 cases per 100 000 population.^4^ Postoperative radiotherapy (RT) is effective at reducing the risk of tumour regrowth after transsphenoidal surgery (TSS).^5^, ^6^ However, patients with NFPA are at significant risk of hypopituitarism, which may be secondary to the tumour itself or a sequela of its treatment. Pituitary irradiation confers a substantial risk of hypopituitarism^7^, ^8^ and has been associated, directly or indirectly, with increased mortality.^9^, ^10^ A causal link between hypopituitarism and mortality has been extensively reported, but many studies are described in the context of syndromes of pituitary hormone hypersecretion, such as acromegaly, Cushing's disease and prolactinoma.^11^, ^12^, ^13^ Functional pituitary tumours introduce the confounding variable of exposure to hormone excess, often associated with adverse cardiometabolic risk, as observed in acromegaly or hypercortisolism.

Such confounders are not applicable in the setting of NFPAs, where hypopituitarism is highly prevalent but where patients have not had cumulative exposure to hormone hypersecretion. Mortality data in NFPA patients with hypopituitarism have been conflicting. A recent large Swedish population‐based study found that excess deaths were observed only in female patients with hypopituitarism.^14^ However, data on the severity of pituitary failure, or on individual pituitary axis deficiencies, were not available, and therefore, independent effects of pituitary axis disruption could not be delineated. An older Danish study of 160 patients with NFPA also found evidence of increased mortality risk in women, but not in men, with hypopituitarism.^15^ The study by Tomlinson *et al*.^16^ included a subset of 573 hypopituitary patients treated for NFPA; only gonadotropin (Gn) deficiency was associated with increased mortality in both sexes.

Irradiation of pituitary tumours, either prophylactically after surgery or reactively after tumour recurrence, also confounds the study of hypopituitarism and mortality. RT itself has been independently associated with increased mortality risk in the treatment of both acromegaly and NFPA,^9^, ^11^ with the higher death rate largely attributable to cerebrovascular disease. However, an independent association between RT and mortality in NFPA has not been consistently replicated across studies, and both the Tomlinson study and the Dutch study of van Varsseveld found no increase in mortality between irradiated and nonirradiated patients.^16^, ^17^ Further confounders in this area include treatment‐associated effects on mortality; overzealous hydrocortisone (HC) replacement in patients with adrenocorticotropic hormone (ACTH) deficiency has been associated with increased mortality, both in NFPA and acromegaly.^11^, ^18^ Growth hormone (GH) and testosterone replacement may reduce mortality in GH and Gn deficiencies, respectively.^16^, ^19^ There are few published data on the impact of levothyroxine (LT4) dosages on mortality in thyroid‐stimulating hormone (TSH) deficiency. Moreover, comprehensive studies on the impact of hypopituitarism and treatment of multiple coexistent hormone deficiencies are sparse in NFPA.

Nonfunctioning pituitary adenomas are an attractive disease model to study these associations, largely due to the ability to exclude the confounding effects of hormone hypersecretion. In this study, we hypothesized that NFPA patients with hypopituitarism have an increased risk of mortality compared to those with normal pituitary function, independent of other potential risk factors such as pituitary irradiation or age at diagnosis; we also hypothesized that treatment of individual hormone deficiencies may itself independently impact on survival. To test these hypotheses, we performed a collaborative European retrospective analysis of a large NFPA cohort, derived from two tertiary referral centres in the UK and Republic of Ireland, and collectively analysed the effects of hypopituitarism on long‐term survival. Patient mortality data were compared across a spectrum of increasing severity of hypopituitarism. We also compared survival data on patients with individual hormone deficiencies to those with intact function of the corresponding pituitary hormone axis. Lastly, we performed a comprehensive assessment of the impact of pituitary hormone replacement on mortality risk. For patients with GH and Gn deficiencies, mortality risk was compared between treated and untreated patients, as a proportion of deficient patients were not on replacement; in the case of HC and LT4 replacement, which is mandatory in ACTH and TSH deficiencies, respectively, the effect of daily replacement doses on relative mortality risk was also examined.

## Materials and methods

### Subjects

Following institutional approval (approval reference CAC‐04929‐12), we performed a retrospective case note analysis of all patients treated for NFPA at University Hospitals Birmingham (UHB), United Kingdom, and Beaumont Hospital Dublin, Republic of Ireland, between 1 January 1999 and 31 December 2014. Both newly diagnosed and existing follow‐up patients reviewed in the Pituitary Clinic for NFPA in both institutions between the above dates were included in the analysis. UHB is a tertiary centre for pituitary disease in the greater West Midlands region of the UK, covering 16 regional referral centres and a total population of 5·7 million. Beaumont Hospital in Dublin is one of only two neurosurgical centres in the Republic of Ireland and receives pituitary referrals from 20 regional hospitals, encompassing a total population of 3·5 million. Data collection was performed by trained staff in both study sites. Mortality data on the Birmingham cohort were obtained via the UHB electronic Clinical Portal, which is linked to both up‐to‐date GP patient records and the Office of National Statistics. Vitality status, date of death and cause of death, where applicable, of patients in Dublin were determined via direct phone contact with the patients' primary care physician.

### Evaluation of pituitary function

Pituitary function testing was performed 2 weeks pre‐operatively, 6 weeks postoperatively and annually thereafter in patients with NFPA treated by surgery and/or RT in both study centres. Baseline pituitary function testing was also performed annually in patients who were treated by observation alone. Overall pituitary function was defined as pituitary reserve at the time of last clinic review before study exit (31 December 2014). GH reserve was not routinely tested in either institution pre‐operatively; postoperative GH deficiency was defined by peak GH levels <3 μg/l on insulin tolerance testing (ITT), or in the absence of ITT (Birmingham patients pre‐2002), by an insulin‐like growth factor‐1 (IGF‐1) level below age and sex‐derived reference ranges in the setting of deficiencies of at least two other pituitary hormones.^20^

A short synacthen test (SST) was performed at UHB pre‐operatively and 6 weeks postoperatively in patients undergoing surgery; a SST was also performed annually in patients who had undergone pituitary RT unless ACTH deficiency was already confirmed. ACTH deficiency in UHB patients was defined as a peak cortisol of <550 nmol/l after 250 μg of intramuscular cosyntropin (tetracosactide), according to locally derived normative data. ACTH reserve in the Dublin patient cohort was assessed by measuring the response of cortisol to either the ITT or the glucagon stimulation test (GST) pre‐operatively and 6 weeks postoperatively, as previously described.^21^ Testing was also performed annually in patients who underwent pituitary RT unless ACTH deficiency was already confirmed. The normal cut‐off, derived from local normative data, was >500 nmol/l for ITT and >450 nmol/l for GST. Due to the high prevalence of a false ‘fail’ rate (approximately 10%) on GST,^22^ subnormal cortisol responses were confirmed by cosyntropin testing (250 μg) in each case (normal >500 nmol/l based on local normative data); patients were only considered ACTH deficient if they failed both tests.

Low levels of free thyroxine (fT4; normal range 10–22 pmol/l) in the presence of low or normal thyroid‐stimulating hormone (TSH; normal range 0·5–4·2 mIU/l), in patients without a pre‐existing history of thyroid disease, were deemed diagnostic of thyrotroph dysfunction in both institutions. Gonadotropin (Gn) deficiency was diagnosed in both institutions as follows: the presence of amenorrhoea, low–normal Gonadotropins and low oestradiol levels (<50 pmol/l) in premenopausal women; follicle‐stimulating hormone (FSH) and luteinizing hormone (LH) levels below normal postmenopausal range (FSH < 30 U/l; LH < 10 U/l, based on locally derived normative reference values) in postmenopausal women; and morning testosterone levels <7 nmol/l (normal range 7·0–27·0 nmol/l) in the setting of low or low–normal FSH and LH levels in men. Permanent diabetes insipidus (DI) was diagnosed as the persistence of hypotonic polyuria, responsive to desmopressin (DDAVP), at the 6‐week postoperative assessment; water deprivation testing was carried out only in selected cases. Multiple hormone deficiencies were defined by deficiency of at least two individual anterior pituitary hormone axes. Panhypopituitarism was defined as the coexistence of GH, Gn, ACTH and TSH deficiencies.

### Statistical analysis

The Statistical Package for the Social Sciences (SPSS, Chicago, IL, USA) version 22 was used for descriptive data analysis. All data are expressed as median and range unless otherwise stated. Independent samples *t‐*tests or Mann–Whitney *U*‐test were used as appropriate for comparison between two groups. One‐way anova with *post hoc* Tukey's testing was used for multiple comparisons between different groups. stata statistical software (StataCorp, College Station, TX, USA, version 14) was used for all mortality analyses. Mortality was analysed using an internal statistical model to avoid the unmeasurable confounders that are likely to exist between the study cohort and the background general population.

A multivariable Cox regression model, with attained age as the time‐scale, and further adjusted for surgery, age at diagnosis, attained age, sex and radiotherapy (RT) exposure, was used to calculate relative risk of death according to pituitary function. RT‐treated patients entered the model for assessment of risk on the date that RT was commenced. Treatment of GH and Gn deficiencies was documented where appropriate, and relative risk was compared to untreated patients with the corresponding hormone deficiency. HC and LT4 dosages were included in the statistical model as time‐ and dose‐dependent variables, as previously described in the context of HC regimens in acromegaly.^11^ Relative risk was calculated for individual hormone deficiencies compared to patients with intact function of the corresponding pituitary axis. We also calculated the relative risk of death across categories of increasing severity of pituitary failure at study exit. *P*‐values were derived from likelihood ratio tests. Kaplan–Meier survival curves for each hormone deficiency were plotted by time since NFPA diagnosis. A two‐sided *P*‐value of <0·05 was considered statistically significant.

## Results

### Patient characteristics

A total of 519 patients with NFPA were included in the study (UHB *n* = 271, Beaumont *n* = 248, 62·2% males, Table 1). Median patient age at diagnosis was 57·0 years (range 18–91). Median length of follow‐up in the combined cohort was 7·0 years (range 0·5–43 years). Birmingham and Dublin patients did not differ significantly with regard to age at diagnosis or duration of follow‐up (*P* = 0·25 and 0·81, respectively). The most common mode of NFPA presentation was visual dysfunction (48·2%), followed by headache/apoplexy (16·5%), incidental (15·6%) and endocrine dysfunction (7·5%), with information on presentation unavailable in 12·2%. On pre‐operative magnetic resonance imaging (MRI) of the pituitary, overt chiasmal compression, cavernous sinus invasion or parasellar extension was documented in 78·8% of patients; only 4·4% of patients had intrasellar tumours. Of 519 patients, 470 underwent surgical debulking of their tumour (TSS 80·8%), with the remainder treated by observation alone; 121 patients (23·3%) had more than one debulking surgery.

**Table 1:** Baseline characteristics of combined patient cohort (*n* = 519) treated for NFPA at University Hospitals Birmingham (UHB) and Beaumont Hospital Dublin between 1999 and 2014. Data presented as number (%) unless otherwise stated. Hormonal status refers to pituitary function at study exit

Treatment centre	
Birmingham	271 (52·2)
Dublin	248 (47·8)
Age at diagnosis (years; median, range)	
Male	57 (18–85)
Female	56 (20–91)
Gender distribution	
Male	323 (62·2)
Female	196 (37·8)
Duration of follow‐up (years; median, range)	
Birmingham	6·0 (0·5–43)
Dublin	7·0 (0·5–35)
Presentation	
Visual disturbance	250 (48·2)
Incidental	81 (15·6)
Headache	48 (9·2)
Endocrine dysfunction	39 (7·5)
Apoplexy	38 (7·3)
Unavailable	63 (12·1)
Treatment strategy	
Surgery	470 (90·5) (TSS *n* = 380; TCS *n* = 90)
Observation alone	49 (9·5)
NFPA immunohistochemistry (*n* = 470 resected tumours)	
Null cell tumour	281 (69·7)
Gonadotrophinoma	74 (18·3)
Silent corticotrophinoma	22 (5·5)
Lactotroph	7 (1·7)
Somatotroph	6 (1·5)
Other/mixed immunostaining	13 (3·3)
Unavailable	67
RT	183 (35·3)
Birmingham	115 (42·4)
Dublin	68 (27·4)
Prophylactic adjuvant RT	80 (43·7)
RT at NFPA recurrence only	103 (56·3)
RT dose (Gy; median, range)	45 (45–50·4)
GH deficiency^a^	325 (65·9)
Gn deficiency^a^	355 (72·0)
ACTH deficiency^a^	318 (64·5)
TSH deficiency^a^	307 (62·3)
Diabetes insipidus^a^	51 (10·3)
HC daily dose (mg; median, range)	20 (5–50)
LT4 daily dose (mcg; median, range)	100 (25–225)
Tumour regrowth	184 (35·4)
Multiple surgeries	121 (23·3)
Deaths	81 (male *n* = 54) (15·6)
Centre	Birmingham *n* = 40; Dublin *n* = 41
Age at death (years; median, range)	78 (24–92)

ACTH, adrenocorticotropic hormone; DI, diabetes insipidus; GH, growth hormone; Gn, gonadotropin; HC, hydrocortisone; LT4, levothyroxine; RT, radiotherapy; TCS, transcranial surgery; TSH, thyroid‐stimulating hormone; TSS, transsphenoidal surgery.

a Complete pituitary function data on 493 patients.

In total, 183 patients (35·3%) received pituitary RT, of whom 181 received conventional three‐field fractionated irradiation. Of the 183 irradiated patients, 80 (43·7%) received prophylactic RT after their first operation; the remainder only received pituitary RT at NFPA recurrence. Median RT dose was 45 Gy (range 45–50·4) administered in a median of 25 fractions (range 25–30). Two patients received stereotactic radiosurgery (SRS). The rate of pituitary RT was higher in Birmingham than in Dublin (42·4% *vs* 27·4%, *P* < 0·001). A total of 289 patients had surgery alone, 181 had surgery plus RT, and 2 patients were treated with primary RT alone. The majority of resected tumours had no stainable immunocontent (null cell tumour, 281 of 403 specimens, 69·7%). Silent gonadotrophinomas (18·3%) and corticotrophinomas (5·5%) constituted the majority of the remaining histological specimens.

In surgically treated patients, extrasellar tumour remnant was observed on postoperative pituitary MRI in 208 of 414 available scans (50·2%). NFPA regrowth was documented in 184 patients (35·4%), with a median time to regrowth of 30 months (range 1–276). Regrowth occurred in 114 of 208 (54·8%) patients with extrasellar tumour remnant and 44 of 131 (33·5%) with intrasellar tumour remnant compared to 6 of 75 (8·0%) patients with no residual tumour or empty sella (*P* < 0·001 for both).

### Endocrine evaluation

The prevalence rates of GH, Gn, ACTH, TSH and antidiuretic hormone (ADH) deficiencies in the total patient cohort at the end of study follow‐up are shown in Table 1. Complete endocrine data were available in 493 patients. The rates of intact pituitary function, single hormone deficiency, multiple hormone deficiencies and panhypopituitarism at study exit were 17·2% (*n* = 85), 9·9% (*n* = 49), 24·9% (*n* = 123) and 47·9% (*n* = 236), respectively. Panhypopituitarism was more common in RT‐treated compared to RT‐naïve patients (56·2% *vs* 43·2%, *P* < 0·001). GH, Gn, ACTH and TSH deficiencies were all significantly more common in irradiated compared to nonirradiated patients (Table 2). Patients treated surgically had a higher rate of panhypopituitarism compared to those managed with observation alone (50·4% *vs* 25·0%, *P* < 0·001). Testosterone was prescribed in all male Gn‐deficient patients unless there was a clear contraindication such as a history of prostate cancer. Oestrogen replacement was given to all premenopausal Gn‐deficient females. Testosterone and oestrogen replacement was prescribed in 79·8% and 21·5% of male and female Gn‐deficient patients, respectively. GH replacement was prescribed for GH‐deficient patients if they met the National Institute of Clinical Excellence criteria (32·3% of GH‐deficient patients).^23^ All ACTH‐ and TSH‐deficient patients were treated with HC and LT4, respectively.

**Table 2:** Characteristics of RT‐ and non‐RT‐treated patients. Data presented as number (%) unless otherwise stated. Hormonal status refers to pituitary function at study exit

	No RT (*n* = 336)	RT (*n* = 183)	*P*‐value
Age at diagnosis (years; median, range)	59 (20–91)	52 (18–79)	<0·001
Duration of follow‐up (years; median, range)	5 (0·5–35)	11 (0·5–43)	<0·001
GH deficiency^a^	199 (62·1)	126 (75)	0·02
Gn deficiency^a^	210 (66·2)	145 (82·3)	<0·001
ACTH deficiency^a^	185 (58·3)	133 (75·5)	<0·001
TSH deficiency^a^	172 (54·1)	135 (76·7)	<0·001
Diabetes insipidus^a^	39 (12·3)	12 (6·8)	0·06
Deaths	51 (15·2)	30 (16·3)	0·40
Age at death (years; median, range)	79 (24–92)	72 (29–91)	0·03

ACTH, adrenocorticotropic hormone; DI, diabetes insipidus; GH, growth hormone; Gn, gonadotropin; TCS, transcranial surgery; TSH, thyroid‐stimulating hormone; TSS, transsphenoidal surgery.

a Complete pituitary function data on 493 patients.

Both pre‐operative and overall (study exit) rates of panhypopituitarism were higher in male compared to female patients (19·5% *vs* 9·1%, *P* = 0·007, and 55·1% *vs* 36·1%, *P* < 0·001, respectively). The age profiles of patients across the spectrum of increasing severity of hypopituitarism (intact, single deficiency, multiple deficiencies and panhypopituitarism) were not statistically significantly different. In surgically treated patients, pituitary function remained unchanged postoperatively in 71·2%, with deterioration of pituitary function observed in 23·3% and improvement in 5·5%.

### Mortality

A total of 81 patient deaths (66·6% male) were recorded in the combined cohort at the end of the study period. The median age at death was 78·0 years (24–92); median time to death from tumour diagnosis was 108 months (0–468). The number of deaths did not differ significantly between men and women, or between Dublin and Birmingham (Table 1). Data on cause of death were available in 70 of 81 patients. Causes of death were categorized as follows: cardiovascular (excluding cerebrovascular, 31·4%), cerebrovascular (22·9%), malignancy (17·1%), infection (17·1%), respiratory (10·0%) and trauma (1·4%). The prevalence rates of GH, Gn, ACTH and TSH deficiencies were higher in patients who later died compared to those still living (*P* < 0·001 for all). Age at death was significantly lower in the RT cohort (*P* = 0·03, Table 2). On internal analysis, after correcting for surgery, age at diagnosis, sex and attained age, RT‐treated patients had a higher relative risk of death than non‐RT‐treated patients (RR 1·62, 95% CI 1·01–2·60, *P* = 0·05, Table 3). On cause‐specific mortality analysis, RT‐treated patients had a higher relative risk of death from infection (RR 2·02, 95% CI 1·03–3·97, *P* = 0·04). There was a trend towards a higher relative risk of cerebrovascular death in the RT group, but this association did not reach significance (RR 4·03, 0·76–21·29, *P* = 0·11). After correction for all associated pituitary hormone deficiencies, RT was not independently associated with excess all‐cause mortality (RR 1·14, 95% CI 0·55–2·37, *P* = 0·73).

**Table 3:** Relative mortality risk (RR) calculated according to individual pituitary hormone deficiencies and radiotherapy (RT) using a Cox regression model

Pituitary function	RR (95% CI)^a^	*P*‐value	RR (95% CI)^b^	*P*‐value
Growth hormone^c^				
Preserved (ref.)				
Deficient	1·09 (0·55–2·16)	0·80	1·17 (0·59–2·33)	0·65
Gonadotropins^c^				
Preserved (ref.)				
Deficient	2·65 (1·14–6·16)	0·01	2·56 (1·10–5·96)	0·01
ACTH^c^				
Preserved (ref.)				
Deficient	2·28 (1·16–4·49)	0·01	2·26 (1·15–4·47)	0·01
TSH^c^				
Preserved (ref.)				
Deficient	1·56 (0·95–2·57)	0·07	1·52 (0·92–2·52)	0·09
Vasopressin^c^				
Preserved (ref.)				
Deficient	0·73 (0·29–1·85)	0·49	0·79 (0·31–2·03)	0·61
Radiotherapy				
No (ref.)				
Yes	1·62 (1·01–2·60)	0·05	–	–
HC daily dose				
0 mg (ref.)				
5–20 mg	2·16 (0·77–6·06)	0·14	2·31 (0·82–6·49)	0·11
21–29 mg	1·63 (0·71–3·76)	0·25	1·79 (0·77–4·15)	0·18
≥30 mg	3·24 (1·30–8·11)	0·01	3·79 (1·49–9·67)	<0·01
*P*‐trend	0·05		0·02	
LT4 daily dose				
0 mcg (ref.)				
<100 mcg	2·44 (1·24–4·79)	0·01	2·41 (1·23–4·73)	0·01
100–150 mcg	1·07 (0·52–2·20)	0·86	1·04 (0·50–2·15)	0·93
>150 mcg	0·62 (0·18–2·12)	0·45	0·61 (0·18–2·09)	0·43
*P*‐trend	0·03		0·03	

ACTH, adrenocorticotropic hormone; HC, hydrocortisone; LT4, levothyroxine; TSH, thyroid‐stimulating hormone. All reference (ref.) RR values = 1·00.

a Internal Cox adjusted for surgery, age at diagnosis, attained age and sex.

b Additionally adjusted for RT.

c Complete pituitary function data on 493 patients.

After correction for surgery, age at diagnosis, sex and attained age, both ACTH and Gn deficiencies were independently associated with increased mortality (RR 2·56, 95% CI 1·10–5·96, *P* = 0·01, and RR 2·26, 95% CI 1·15–4·47, *P* = 0·01, respectively, Table 3). Relative risk of death was increased in those ACTH‐deficient patients taking total daily hydrocortisone doses of 30 mg or higher (RR 3·79, 95% CI 1·49–9·67, *P* < 0·01, *P*‐trend = 0·02, Table 3). Further subanalysis of Gn deficiency according to gender, after additional adjustment for dysfunction of all other pituitary hormone axes, revealed a significant association with increased mortality in male patients only (RR 5·44, 95% CI 1·07–27·69, *P* = 0·02). Male patients with untreated hypogonadism had a trend towards a higher relative risk of death than their treated counterparts, but this comparison did not reach statistical significance (Table 4). No significant mortality difference was observed between treated and untreated Gn‐deficient women.

**Table 4:** Relative mortality risk in treated (Ref. 1·00) and untreated gonadotropin and GH deficiency

Pituitary function	RR (95% CI)	*P*‐value
Gonadotropin deficiency^a^		
Male		
Treated	1·00	
Untreated	1·72 (0·71–4·16)	0·22
Female		
Treated	1·00	
Untreated	0·21 (0·03–2·77)	0·62
GH deficiency^a^^,^ b		
Treated	1·00	
Untreated	5·81 (0·73–46·23)	0·04

a Complete data on 493 patients. Internal Cox adjusted for surgery, RT, age at diagnosis and attained age.

b Additionally adjusted for sex.

**Figure 1:**
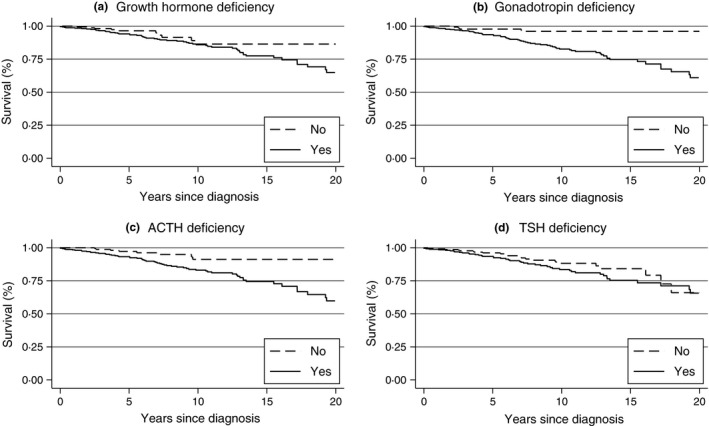
Kaplan–Meier curves (unadjusted) in the combined Dublin and Birmingham cohort according to hormonal status at study exit (a) Growth hormone (GH) deficiency, (b) gonadotropin (Gn) deficiency, (c) adrenocorticotropic hormone (ACTH) deficiency and (d) thyroid‐stimulating hormone (TSH) deficiency. After adjustment, independent effects on increased mortality were observed only in ACTH and Gn deficiencies at study exit.

Although there was only a trend towards increased mortality in TSH‐deficient patients, relative risk of death was increased in those patients taking <100 mcg daily dose of levothyroxine (RR 2·41, 95% CI 1·23–4·73, *P* = 0·01, Table 3). This association disappeared at doses above 100 mcg daily (*P* = 0·86 and *P* = 0·45 for dosages ≥100 and >150 mcg, respectively, *P*‐trend = 0·03). GH‐deficient patients were not at overall increased risk of death compared to those with intact GH secretion (Fig. 1). However, when considered separately, mortality risk was higher in untreated compared to treated GH deficiency (Table 4).

There was a statistically significant linear trend towards increased mortality risk across a spectrum of severity of pituitary failure, with the highest risk observed in those with panhypopituitarism (*P*‐trend = 0·04, Table 5).

**Table 5:** Relative mortality risk (RR) according to severity of hypopituitarism at study exit using a Cox regression model^b^

Severity of pituitary dysfunction	No. of deaths	RR (95% CI)^a^	*P*‐value
Normal pituitary function (ref.)	2/85	1·00	–
Single hormone deficiency	4/49	2·27 (0·39–13·05)	0·36
Multiple hormone deficiencies	23/123	3·13 (0·71–13·86)	0·13
Panhypopituitarism	45/236	3·58 (0·84–15·24)	0·08

a *P*‐trend = 0·04.

b Calculated in 493 patients with complete pituitary function data at study exit. Internal Cox adjusted for surgery, RT, age at diagnosis, attained age and sex.

## Discussion

In this large cross‐sectional study, we have performed a comprehensive analysis of the association between hypopituitarism and mortality in NFPA. We have also examined the impact of hormone replacement on relative risk of death; in the case of HC and LT4, we have performed an in‐depth analysis of the effect of different daily replacement dosages on mortality. To our knowledge, this is the first international collaborative study of mortality in patients treated for NFPA, combining long‐term follow‐up data from two large European cohorts, with a total patient number in excess of 500. Internal statistical modelling has identified ACTH and Gn deficiencies as conferring greater than a two‐fold increased relative risk of death compared to those with intact axes. The vast majority of patients had detailed annual pituitary function testing over a median follow‐up period of 7 years; many patients were followed for much longer. Comprehensive data on mortality status, tumour presentation, surgery, histology, RT and hormone replacement were also available on all patients. Our data convincingly reinforce the dogma that risk of death increases in HC‐treated patients in a dose‐dependent fashion; conversely, patients with secondary hypothyroidism may be chronically untreated, potentially contributing to adverse cardiometabolic risk. We believe that NFPA is the ‘purest’ biological model of pituitary disease within which to consider the association between hypopituitarism and mortality, due to the exclusion of secretory pituitary tumours. Furthermore, by utilizing an internal statistical analysis, we have attempted to avoid the potential confounding effect of unquantifiable variables that differ between the study cohort and the background population.

There has been extensive interest in the subject of hypopituitarism and mortality in the last 25 years. Rosen and Bengtsson^24^ first documented premature mortality due to cardiovascular disease in a seminal publication in 1990. The study by Tomlinson found a standardized mortality ratio (SMR) of 1·87 in hypopituitary patients compared to the external population.^16^ However, all patients in this study had hypopituitarism of varying severity, and so comparison with an internal control group with normal pituitary function was not possible. In our unselected cohort of consecutive patients with NFPA, we found a highly significant statistical linear trend between the severity of pituitary failure and risk of premature death, with the highest risk observed in those with panhypopituitarism. We included all NFPA patients in the analysis, including a cohort of 49 patients who were managed with observation alone. Both ACTH and Gn deficiencies predicted mortality in our study. We did not find a significant association between GH, TSH or vasopressin deficiencies and risk of death in our NFPA cohort. However, when analysed separately, patients with treated GH deficiency clearly had a survival benefit over their untreated counterparts, with a fivefold higher relative mortality risk in the untreated group. Adult‐onset GH deficiency has been associated with increased mortality in some but not all studies;^16^, ^19^, ^25^ of these studies, van Bunderen *et al*.^19^ found that treatment with GH restored cardiovascular risk to normal in male, but not in female, patients. Our data also support our hypothesis that the cumulative effect of dysfunction of multiple hormonal axes may confer an increased relative risk of death, even in the absence of independent contributions from individual axes. This observation may be attributable to factors such as inadequate or overzealous hormone replacement, medication interaction or other as yet undefined physiological phenomena.

Our observation of increased mortality in ACTH‐deficient NFPA patients mirrors data from patients with acromegaly, and in other cohorts with pituitary dysfunction.^11^, ^26^ ACTH deficiency leads to hypocortisolism during acute illness,^27^ with a significant risk of life‐threatening adrenal crisis. It is also intimately associated with the adverse metabolic effects of chronic supraphysiological glucocorticoid replacement.^18^, ^28^ We have shown that mortality risk is increased almost fourfold in patients taking total daily hydrocortisone doses of 30 mg or higher. Zueger *et al*.^18^ also found increased mortality with higher hydrocortisone dosages in ACTH‐deficient NFPA patients in a Swiss study; this finding was consistent for both total and weight‐adjusted hydrocortisone doses, and the same associations have been observed in acromegaly.^11^ Interestingly, we found that daily replacement doses of LT4 below 100 mcg in TSH‐deficient patients were associated with a higher mortality risk. To our knowledge, this unexpected observation has not been demonstrated previously in secondary hypothyroidism. Optimal treatment of TSH deficiency is limited by the absence of a reliable biomarker of adequate replacement, as LT4 doses cannot be titrated according to TSH levels in pituitary failure. These data support the clinical suspicion that many pituitary patients with secondary hypothyroidism are chronically undertreated.^29^

Gonadotropin deficiency conferred the highest relative risk of death of all pituitary axis deficiencies in our study. This finding mirrors results from the Tomlinson study, where untreated Gn deficiency in the NFPA subgroup was associated with an increased risk of death (SMR 2·85); this risk was normalized by hormone replacement.^16^ Subgroup analysis in our Gn‐deficient cohort found that this association was only significant in men; hypogonadal males had a greater than fivefold increased risk of mortality compared to the eugonadal reference cohort. Male hypogonadism is associated with dyslipidaemia, hypertension, insulin resistance and atherosclerosis,^30^, ^31^ but it remains divisive whether is an independent risk factor or simply a surrogate marker for underlying metabolic disease.^32^ Low testosterone is also associated with increased mortality in unselected cohorts without evidence of pituitary disease.^33^, ^34^ In our study, hypogonadal men who were not treated with testosterone had a trend towards a higher relative risk of death; however, the statistical power of this analysis is likely to be limited by relatively small numbers of untreated hypogonadal men (21·5%). Equally, no mortality benefit or risk was conferred by oestrogen replacement in our study, but, once again, this analysis is likely to be underpowered; only 20% of Gn‐deficient females were treated with oestrogen, and only a very small number of deaths were observed in premenopausal females.

Other mortality studies in NFPA patients with hypopituitarism have shown conflicting results to date. A recent study of 546 patients from Oxford found increased overall mortality on external analysis (SMR 3.8) in a series of 546 patients operated for NFPA.^35^ Only young age at diagnosis was predictive of premature death on multivariate analysis, with no independent contribution from individual pituitary hormone deficiencies. However, the study did not include patients with microadenomas, and only included surgically treated patients, which is likely to reduce the proportion of patients with normal pituitary function to include as an internal comparator group. Another Swedish population‐based study of over 2700 patients with NFPA recently showed premature mortality only in female NFPA patients with hypopituitarism or diabetes insipidus.^14^ The overall SMR for patients with hypopituitarism was similar to the background population, but detailed data on pituitary function were not available to the authors.

Patients who underwent pituitary irradiation in our study had a significantly increased risk of death (RR 1·62). The median age at death in RT‐treated patients was 7 years younger than in the RT‐naïve cohort. However, after correction for pituitary dysfunction, this association was no longer significant, suggesting that the observed increase in premature mortality is mediated by RT‐induced hypopituitarism rather than an independent effect of RT itself. These data are consistent with findings from Tomlinson and other older studies,^16^, ^36^ as well as the more recent van Varsseveld and Oxford studies.^17^, ^35^ There was a trend towards an increased risk of cerebrovascular death compared to nonirradiated patients, but this did not reach statistical significance. Intriguingly, RT‐treated patients had a twofold increased risk of death from infection. This association is likely to be mediated by RT‐induced hypopituitarism. This is particularly true in those with ACTH deficiency, who are at significantly increased risk of succumbing to infection‐induced adrenal crisis.^37^ Whether RT is independently associated with premature mortality, or whether these associations are driven purely by hypopituitarism, will remain a matter of debate. It is increasingly clear, however, that conventional three‐field fractionated pituitary RT is associated, directly or indirectly, with morbidity and mortality. Long‐term data on SRS are sparse, however; recent studies suggest reduced neurotoxicity, although the prevalence of hypopituitarism may still be relatively high.^38^

Our study has a number of limitations. Pituitary function data, as in most studies on hypopituitarism, are presented as a snapshot at study exit, rather than as a dynamic variable throughout follow‐up. Secondly, there is a degree of heterogeneity in the evaluation of pituitary function between the two centres. In Birmingham, a cosyntropin test was used for assessment of ACTH reserve, while Dublin patients underwent ITT, or GST followed by cosyntropin test where appropriate. While controversy exists over the most appropriate test for secondary adrenal insufficiency in pituitary disease, recent data suggest good correlation between distinct testing methods, as long as unique normative cut‐off levels have been determined for each (as was the case in both study centres).^39^ A policy of dynamic testing of GH reserve was not universally applied to the Birmingham cohort pre‐2002. However, IGF‐1 levels below age and sex‐derived reference ranges, in the setting of deficiencies of at least two other pituitary hormones, have been validated as a reasonable diagnostic alternative in cases where ITT is not available.^20^, ^40^ Although desirable, complete homogeneity of dynamic testing strategies is not realistic when combining pituitary function data from different centres. These limitations notwithstanding, we believe that pituitary function and mortality data have been accurately collated on all patients in both cohorts. The predominant strength of this study lies in the size of the population studied, which comprises in excess of 500 patients. The novelty of our work is underpinned by the combination of in‐depth phenotyping of pituitary function with comprehensive data on treatment of multiple pituitary hormone deficiencies. This has facilitated robust analysis of the associations between hypopituitarism, under‐ or overreplacement of deficient hormones, and, ultimately, risk of death.

In summary, in this large cohort of 519 NFPA patients derived from tertiary neurosurgical centres in the UK and Republic of Ireland, we have identified independent associations of both ACTH and Gn deficiencies with mortality. Whether individual hormone deficiencies themselves, or more complex treatment‐associated factors, underpin these relationships requires further work, particularly in the context of male hypogonadism. Excessive HC replacement, coupled with suboptimal treatment of central hypothyroidism, may contribute further to the adverse cardiometabolic milieu in hypopituitary NFPA patients. Whilst pituitary RT is also associated with an increased relative risk of death in NFPA, the connection with increased mortality may be attributable simply to the high prevalence of hypopituitarism in irradiated patients. We have also shown that mortality risk in patients treated for NFPA increases in a linear fashion across a spectrum of pituitary failure. This suggests a cumulative contribution of multiple hormone deficiencies, which is potentially greater than the sum of individual risk.
